# Predictive clinical and hematological factors in the differential diagnosis between malignancy and arthritis in children: a case-control study

**DOI:** 10.1186/1546-0096-9-S1-P236

**Published:** 2011-09-14

**Authors:** Cristina Trigilia, Martina Barchitta, Tiziana Timpanaro, Silvia Marino, Rosaria Garozzo, Manuela La Rosa, Giovanna Russo, Antonella Agodi, Patrizia Barone, Andrea Di Cataldo

## Introduction

Musculoskeletal pain is a common symptom in childhood and in a few cases is the initial sign of a severe disease such a chronic inflammatory disease or a malignancy.

## Aim

To identify the predictive factors for malignancies in children presenting with musculoskeletal pain, and ultimately diagnosed to have JRA or malignancy.

Patients and methods: it is a retrospective case-control chart review study examining data from the initial visit of all the patients referred to our Department for musculoskeletal pain during the period January 2001–December 2010 and then diagnosed with a tumour or with JRA. The value of given diagnostic test as a predictive factor for neoplasia in comparison to JRA, were assessed by calculating its sensitivity, specificity, positive predictive value, negative predictive value.

## Results

No significant difference was found for sex, age and pattern of pain. Fever and lymphadenopathy were more frequent in children with neoplasia. Elevation of CRP and of ESR and an abnormal platelet count were not relevant parameters. Anaemia and an abnormal neutrophil count were most frequent among patients with tumours.

High LDH level presents with a higher frequency and a statistically significant difference (sensitivity 88% and specificity 32.6%) among the tumours group and resulted a useful predictive factor for tumours, with high sensitivity.

## Conclusions

Our results suggest to take into great account even “soft” changes in the neutrophil count particularly if it is associated to a low Hb level, and to evaluate LDH that, if associated to other abnormal laboratory data should require to go thoroughly into the analysis to always exclude a malignancy.
